# Enhancing the Hydrolytic Stability of Poly(lactic acid) Using Novel Stabilizer Combinations

**DOI:** 10.3390/polym16040506

**Published:** 2024-02-13

**Authors:** Jannik Hallstein, Elke Metzsch-Zilligen, Rudolf Pfaendner

**Affiliations:** Fraunhofer Institute for Structural Durability and System Reliability LBF, Division Plastics, 64289 Darmstadt, Germany; jannik.hallstein@lbf.fraunhofer.de (J.H.); elke.metzsch-zilligen@lbf.fraunhofer.de (E.M.-Z.)

**Keywords:** poly(lactic acid), hydrolytic degradation, aziridines, hydrolysis inhibitors, acid regulators

## Abstract

Commercially available poly(lactic acid) exhibits poor hydrolytic stability, which makes it impossible for use in durable applications. Therefore, a novel hydrolysis inhibitor based on an aziridine derivative as well as a novel stabilizer composition, containing an aziridine derivative and an acid scavenger, were investigated to improve the hydrolytic stability. To evaluate the stabilizing effect, the melt volume rate (MVR) and molecular weight were monitored during an accelerated hydrolytic aging in water at elevated temperatures. Temperatures were selected according to the glass transition temperature (~60 °C) of PLA. It was shown that the novel hydrolysis inhibitor as well as the novel stabilizer composition exhibited excellent performance during hydrolytic aging, exceeding commercially available alternatives, e.g., polymeric carbodiimides. A molecular weight analysis resulted in a molecular weight decrease of only 10% during approximately 850 h and up to 20% after 1200 h of hydrolytic aging, whereas poly(lactic acid) stabilized with a commercial polycarbodiimide revealed comparable molecular weight reductions after only 300 h. Furthermore, the stabilization mechanism of the aziridine derivative alone, as well as in the synergistic combination with the acid scavenger (calcium hydrotalcite), was investigated using nuclear magnetic resonance (NMR) spectroscopy. In addition to an improved hydrolytic stability, the thermal properties were also enhanced compared to polymeric carbodiimides.

## 1. Introduction

Due to their petro-based raw material sources, most plastics are increasingly falling into disrepute in public perception. This is especially true for plastics in the packaging sector, but the reputation of plastics in technical applications also suffers. Therefore, material manufacturers, compounders and processors are challenged to present new solutions to counteract this trend. One possibility here is the use of bio-based plastics such as poly(lactic acid) (PLA), poly(butylene succinate) (PBS) or polyhydroxyalkanoates (PHA) [1,2,3,4]. However, due to their low long-term durability, these materials are currently primarily used in the packaging sector or for disposable articles, with the focus mostly on the biodegradability of the polymer in order to address the waste problem in the environment [5,6,7,8]. From the viewpoint of the circular economy, however, this is the most unsatisfactory solution, since, here, the cycle from metabolization to renewed monomer or polymer synthesis is the most prolonged. In addition, the previously bound CO_2_ is released again, which significantly worsens the ecological balance [9]. A more direct route from the end of life to a new product, for example, through mechanical recycling, would be preferable [10,11,12,13,14,15]. The existing areas of application for bioplastics are only suitable for this purpose to a limited extent, as they only take up a small amount compared to petro-based plastics, and sorting and recycling them is not economically viable today [16]. Better suited are materials from more durable applications that can be recycled in a closed loop and do not end up in the general stream of plastic packaging [17,18]. Due to its mechanical properties, high strength and stiffness, PLA is the bioplastic most suitable for long-term technical applications [19,20,21]. The issue with PLA, however, is its sensitivity to hydrolysis and its slow crystallization behavior, which results in a low long-term operating temperature [22]. Due to the fact that most commercially available bio-based plastics are aliphatic polyesters, they are sensitive to thermo-hydrolytic degradation, causing the polymers to degrade rapidly and lose their properties [23,24,25,26,27]. As a result, these materials are not yet suitable for durable applications or for mechanical recycling.

**Figure 1:**
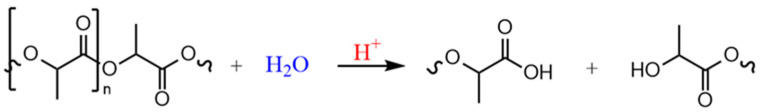
Acid-catalyzed hydrolytic degradation mechanism of poly(lactic acid).

The focus of this work was, therefore, on improving the hydrolytic resistance of PLA. For this purpose, various additive systems were compounded into PLA and evaluated. However, in order to optimally select the components of these systems, the specific degradation mechanism and the various factors influencing it are relevant. As PLA is an aliphatic polyester, hydrolytic chain scission is the main degradation mechanism (shown in Figure 1), which occurs randomly along the polymer backbone [24].

The moisture enters the polymer matrix via diffusion processes from the environment. Usually, polyesters exhibit carboxyl end groups which have an autocatalytic effect [25,26]. Furthermore, a catalyzing effect occurs due to the intrinsic dissociation of the absorbed moisture. For an efficient hydrolysis stabilization, an additive system that optimally acts on both factors must, therefore, be designed. One class of additives that reacts with moisture and the carboxyl end groups is defined as hydrolysis inhibitors. Primarily, carbodiimides are used here, which have already been studied many times in PLA [28,29,30,31,32,33,34,35,36]. They are commercially available in various structural configurations (monomeric, polymeric, aliphatic, aromatic, cyclic). Porfyris et al. [28] investigated various carbodiimide types, showing that in semi-crystalline PLA, the polymeric carbodiimides, in particular, have a good stabilizing effect. However, a problem with the stabilization through carbodiimides or inhibitors, in general, is that they are consumed due to their direct reaction with the penetrating moisture and must be added in high concentrations. Stloukal et al. [29] were able to observe a significantly prolonged hydrolytic stability only from an additive content of >1.25%. The high additive content makes the compounds uneconomical, as the carbodiimides are comparatively expensive. Due to this, in this work, an alternative inhibitor was investigated. However, it can be assumed that a stabilizing effect is only achieved in the case of a direct reaction with the moisture and the carboxyl groups. Additives that react only with the carboxyl end group, but not with moisture, e.g., epoxy-based additives, exhibit no effect [28,36]. Aziridines are known to react with water at elevated temperatures [37,38], but aziridine-based structures have, so far, only been investigated in PLA as crosslinkers for star or long-chain branching [39] but not for their effect on hydrolysis resistance. Therefore, the present study investigated a novel approach with aziridine-based additives as hydrolysis inhibitors in polylactic acid.

Another strategy to alter the degradation catalyst H^+^ is via the modification of the pH value. Various studies have demonstrated that the degradation rate of PLA is influenced by the pH of the storage medium [40,41,42,43]. Since changing the pH of moisture entering the polymer is not possible in real applications, this work incorporated additives that regulate the pH in the polymer matrix into PLA. Acid scavengers, which are already used in other polymers (e.g., PP or PVC) for stabilization, are suitable additives for this case [44]. These include oxides, carbonates of alkali metals, hydrotalcites or alkali metal salts of phosphates. The fact that the degradation rate of PLA in in vivo applications can be influenced by the addition of acid-regulating substances has already been demonstrated in previous work [45,46,47]. Ara et al. [48] also investigated PLGA co-polymers with different calcium-based acid scavengers and found a reduced degradation rate with increased basicity, although 30% of the acid scavenger was added. Therefore, the high concentrations significantly reduced the bio-based contents in the compound. A positive effect due to hydrotalcites was also described by Valentina et al. [49,50], even though the stabilization effect was attributed to improved crystallinity.

Considering both stabilization concepts, in the presented experiments, we developed and investigated an additive system that makes use of both mechanisms and, thus, results in a synergistic stabilizing effect.

(1) An alternative hydrolysis inhibitor to the carbodiimides was tested, and its stabilization mechanism was investigated, which formed the base of the additive system.
(2) A synergistic co-stabilizer, based on an acid-scavenger, was added, which not only slowed down the degradation reactions of the polymer chains with moisture, but also protected the inhibitor from an accelerated reaction with moisture, delaying the consumption of the hydrolysis inhibitor.

The use of an acid-scavenger as a co-stabilizer was investigated by Polidar et al. [51]. There, however, it was added to alter the effectiveness of a degradation catalyst and to control accelerated degradation. As a co-stabilizer in a system for improved hydrolysis stabilization, no studies are known, to the best of our knowledge.

## 2. Materials and Methods

### 2.1. Materials

A commercially available PLA grade (Luminy L130, TotalEnergies Corbion, Gorinchem, The Netherlands) was used. This grade is a fully biobased semicrystalline PLLA grade with an l-isomer content of at least 99%. The high isomeric purity enables a good crystallization behavior and its use in high-heat applications. As a hydrolysis inhibitor, different commercially available additives were used: As state-of-the-art reference monomeric carbodiimide (mCDI) (Stabilisator 7000, Raschig, Ludwigshafen am Rhein, Germany) and polymeric carbodiimide (pCDI) (Stabaxol P, Lanxess, Cologne, Germany) were used. As a novel hydrolysis inhibitor a bifunctional aziridine derivate (PolyU) (PolyU, Menadiona, Barcelona, Spain) was used. As an acid scavenger, an uncoated calcium hydrotalcite (HTC) (Actilox CAH EXP 0213, Nabaltec, Schwandorf, Germany) was used.

### 2.2. Compounding

Compounding was performed on a co-rotating parallel twin-screw extruder Process 11 (Thermo Fisher Scientific, Karlsruhe, Germany) with a screw diameter of 11 mm and an L/D ratio of 40. Prior to the compounding, PLA granules were cooled in liquid nitrogen and milled on a cutting mill (Rapid Granulier Systeme GmbH & Co.KG, Kleinostheim, Germany). Before the extrusion process, milled PLA was vacuum-dried for 16 h at 80 °C to a moisture content of below 250 ppm. The calcium hydrotalcite was vacuum-dried for 16 h at 150 °C. Stabilisator 7000, Stabaxol P and PolyU were used as delivered. Milled polymer and additives were premixed in a bag and added to a volumetric dosage unit, which was set to a mass throughput of 800 g per hour. The screw speed of the extruder was set to 200 rpm, and the temperature profile was set to increase from 170 °C in the feeding zone to 200 °C in the mixing zones and the die. Vacuum degassing, a water bath and a pelletizer were used.

### 2.3. Hydrolytic Aging Tests

For hydrolytic aging tests, around 20 g of compounded PLA granules were added to a glass jar (200 mL). Deionized water was added to fill the jars completely. The jars were closed with a lid and placed in a heating chamber with forced convection (Binder M115, Binder GmbH, Tuttlingen, Germany), which was set to an aging temperature of 60 °C. The temperature was selected to be in the glass transition temperature range of polylactic acid (~60 °C). The jars were removed in different time intervals, and after each removal, the granules were dried in a vacuum oven at room temperature for at least two hours and at 80 °C for 16 h. After drying, the granules were sealed in vacuum bags until further testing. At the beginning and at every removal time, the pH of the aging medium was monitored with a pH meter (FiveEasy Plus™ pH FP20, Mettler-Toledo GmbH, Gießen, Germany).

### 2.4. Characterization

#### 2.4.1. Melt Flow Index (MFI)

The melt volume rate (MVR) was measured on a mi2 melt flow indexer (Göttfert Werkstoff-Prüfmaschinen GmbH, Buchen, Germany) according to DIN EN ISO 1133-2 [52]. The temperature was set to 190 °C, and the stamp was loaded with a weight of 2.16 kg. The heating time was set to four minutes, and about 8 g of granules were used to perform the tests. Prior to MVR measurements, the pellets were dried in a vacuum oven at 80 °C for 16 h and sealed in vacuum bags until shortly before the start of the measurement to ensure no further hydrolysis during the heating time. For each removal time, two measurements per compound were performed, the average value of both experiments is reported below.

#### 2.4.2. Size Exclusion Chromatography (SEC)

Size exclusion chromatography (SEC) measurements were performed using a SEC 1260 system by Agilent Technologies (Waldbronn, Germany) consisting of a degasser (G1322A), isocratic pump (G1310B), autosampler (G1329B), thermostat (G1316A), variable wavelength detector (G1314F), refractive index detector (G7800A), two Agilent-PLgel-MIXED-C columns and PLgel guard column. Chloroform was used as the eluent (c = 2 g L^−1^) at 35 °C with a flow rate of 1 mL min^−1^. Calibration was performed using a polystyrene standard (PSS Polymer Standards Service GmbH, Mainz, Germany) over a molecular weight distribution of 417–2,520,000 g mol^−1^.

#### 2.4.3. Spectroscopic Characterization (NMR, FTIR)

Nuclear magnetic resonance spectroscopy (NMR) was performed on a Bruker NanoBay 300 spectrometer (7.05 T, Bruker, Ettlingen, Germany). To investigate the reaction of the PolyU with moisture during the hydrolytic aging, approximately 15 mg of PolyU was weighed in an NMR tube and filled with 0.6 mL DMSO-d6 and 0.15 mL H_2_O at two different pH values (7.0 and 2.1). The pH value of the H_2_O was reduced by adding 10% of a 90 Vol% lactic acid solution purchased from SigmaAldrich (Taufkirchen, Germany). The tubes were placed in the spectrometer for 24 h, while being heated up to 60 °C. Every 30 min, an ^1^H-NMR spectrum was recorded to track the reaction of PolyU with H_2_O.

To investigate the reaction of the PolyU with carboxylic acid groups, approximately 15 mg of PolyU and 15 mg of stearic acid, purchased from ThermoScientific (Schwerte, Germany), were weighed in an NMR tube and filled with 0.7 mL DMSO-d6. The tubes were placed in a convection oven (Binder M115, Binder GmbH, Tuttlingen, Germany) for 15 min at 150 °C to ensure reaction. Before and after oven storage, ^1^H-NMR spectra were recorded.

Fourier-transform infrared spectroscopy (FTIR) spectra were recorded by using a Nicolet 8700 FTIR spectrophotometer with a Golden Gate ATR (Thermo Fisher Scientific, Waltham, MA, USA). 32 scans were performed for every spectrum. The spectral resolution was 4 cm^−1^, while the measurements were recorded in a range between 4000 and 650 cm^−1^.

#### 2.4.4. Differential Scanning Calorimetry (DSC)

To determine the thermal properties of the compounds, differential scanning calorimetry (DSC) was performed on a DSC 822e (Mettler Toledo GmbH, Gießen, Germany). Approximately 15 mg of PLA granules were weighed in an aluminum crucible. Samples were heated up to 200 °C and cooled down to 10 °C with heating/cooling rates of ±10 K min^−1^ under N_2_ flow. This was performed twice for each sample. The crystallinity (X_c_) was determined by relating the enthalpy of fusion (ΔH_m_) to the enthalpy of fusion of a 100% crystalline polymer (ΔH_m0_ = 93 J g^−1^) [53,54,55]. Glass transition temperature (T_g_) was determined at the midpoint of transition.

## 3. Results

### 3.1. Hydrolysis Behavior and Characterization of PLA Compounds Stabilized with a Hydrolysis Inhibitor

**Figure 2:**
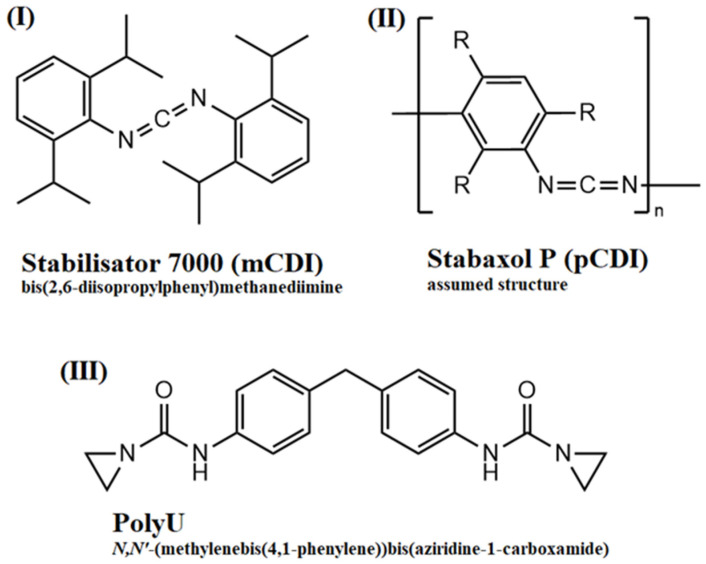
Chemical structures of used hydrolysis inhibitors: (**I**) monomeric carbodiimide; (**II**) polymeric carbodiimide; (**III**) aziridine-based inhibitor.

The hydrolytic degradation of poly(lactic acid) and of the compounds stabilized with hydrolysis inhibitors was quantified through measurements of the MVR during the hydrolytic aging of compounded granules at an aging temperature of 60 °C. The temperature was chosen to be in the range of glass transition temperature of PLA, which led to the faster diffusion of moisture into the sample and accelerated aging. Samples were taken before aging and after four different aging times (2, 4, 7 and 14 days). Measurement of MVR provided information on the melt viscosity of the samples and can, therefore, be used as an indicator of polymer chain degradation. The higher the MVR, the lower the melt viscosity and, thus, the molecular weight. Therefore, strongly increasing MVR values are an indicator for severe chain degradation. The chemical structures of the hydrolysis inhibitors used are shown in Figure 2.

**Figure 3:**
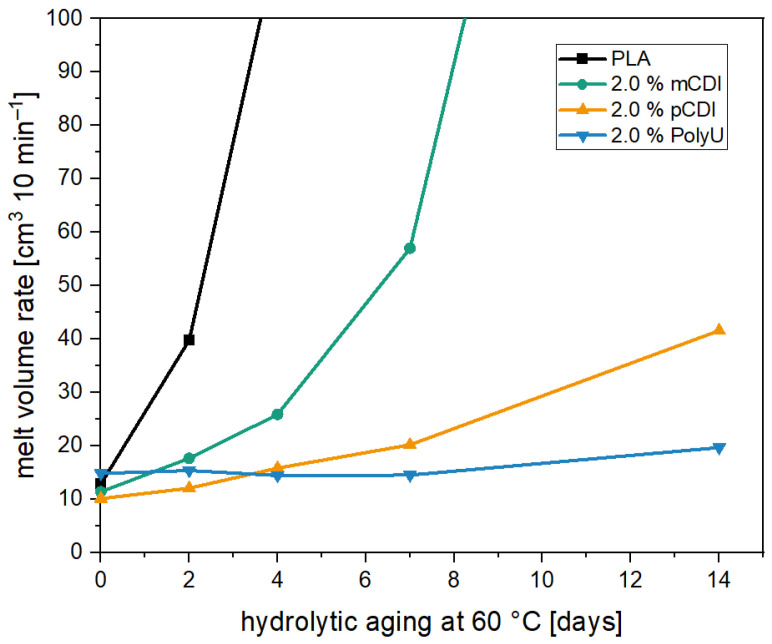
MVR values of aged PLA samples unstabilized (black) and stabilized with three different hydrolysis inhibitors over the course of 14 days at a temperature of 60 °C.

Figure 3 shows the MVR values during hydrolytic aging. The observation of the MVR values demonstrates the expected rapid increase for the unadditivated PLA. After only two days, the MVR increases by 400% from 10 cm^3^ 10 min^−1^ to over 40 cm^3^ 10 min^−1^. After two more days, the MVR increases by another 300%. The sharp increase in MVR values is equivalent to a reduction in melt viscosity and is in line with expectations. The increased temperature accelerates the diffusion of moisture into the material, and the hydrolytic ester cleavage takes place due to the lack of stabilization. This leads to a fast reduction in the molecular weight, which is further accelerated by newly formed carboxyl end groups, which have an autocatalyzing effect [24,25,26]. After further aging, the MVR could not be measured due to the low viscosity of the material. The investigation of the molecular weight of the unstabilized PLA reveals a decrease in molecular weight by about 90%, with the number-average molecular weight being below 5000 g mol^−1^. Before aging, the number-average molecular weight lies above 50,000 g mol^−1^. Similar molecular weight reductions have also been observed in other studies during aging at similar conditions [29,51]. This confirms that the selected temperature is suitable for accelerated aging, and the hydrolysis inhibitors can be evaluated. For this purpose, 2% of each hydrolysis inhibitor was compounded into PLA, and the samples were subjected to water storage. The additive dosage was selected based on previous work with monomeric carbodiimide (mCDI), which showed that significant stabilization only occurred from 1.5%, with the highest values at 2.0% [29].

The addition of the mCDI, however, resulted only in a slight stabilization of the polymer chains against hydrolytic degradation. After only four days, the MVR value was more than doubled (11.4 cm^3^ 10 min^−1^ → 25.9 cm^3^ 10 min^−1^), with further doubling after seven days. After 14 days, the sample had already degraded to such an extent that no reliable value could be measured. The polymeric carbodiimide, on the other hand, showed a significantly higher stabilization effect during aging. The doubling of the MVR values occurred here only after 7 days of water storage, followed by a further doubling after 14 days at the end of the accelerated aging (10.1 cm^3^ 10 min^−1^ → 20.2 cm^3^ 10 min^−1^ → 41.6 cm^3^ 10 min^−1^). After 14 days, the values were at a level that was reached for the unstabilized PLA after only two days.

A molecular weight analysis confirmed the significantly improved stabilizing effect by the addition of the selected inhibitors. Thus, the reduction of the number average molecular weight for the samples with 2.0% pCDI was only 25%. This is consistent with other studies in which carbodiimides were used for stabilization [28,29,30,31,32,33,34,35,36]. In some cases, however, even lower molecular weight reductions were observed during hydrolytic aging [29]. The low stabilization effect of the monomeric carbodiimide is surprising. Due to the chemical structure, both carbodiimides should react with the diffusing moisture and the carboxyl end groups of the PLA, as previously described in the literature, and investigated using FTIR spectroscopy [28,32]. Although this phenomenon was previously described for semicrystalline poly(lactic acid), as also used in this study, by Porfyris et al. [28].

However, the longest-lasting stabilizing effect was observed with the addition of the aziridine-based inhibitor (PolyU). Although processing led to a slight increase in MVR (12.8 cm^3^ 10 min^−1^ (PLA) → 14.8 cm^3^ 10 min^−1^), the hydrolysis-stabilizing effect did not seem to be negatively affected. During accelerated aging, it was seen that within the first week, there was still no increase in the melt volume flow rate. Even after seven more days, only a minimal increase was observed (14.8 cm^3^ 10 min^−1^ → 19.7 cm^3^ 10 min^−1^). The very good stability of the samples was also demonstrated by determining the molecular weight. The number-average molecular weight was reduced by only 10% and was, thus, once again, significantly lower than the reduction of the samples with a polymeric carbodiimide. Since the aziridine-based additive has a functional unit, and these structures are usually used as crosslinkers, it is assumed that the molecule can also react directly with the moisture migrating into the polymer as well as the carboxyl end groups of the PLA, as it has been demonstrated for the carbodiimides.

**Figure 4:**
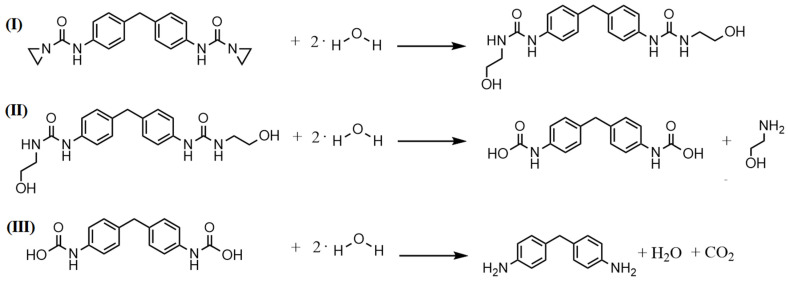
Proposed reaction mechanism of the aziridine-based hydrolysis inhibitor with moisture (**I**) and the further reaction of the resulting reaction products (**II** + **III**), therefore, protecting the polymer chain from hydrolytic degradtion.

**Figure 5:**
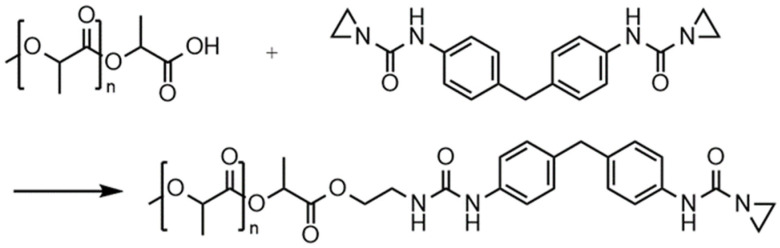
Reaction mechanism of aziridine-based hydrolysis inhibitor with carboxyl end groups of poly(lactic acid), which, therefore, inhibits the autocatalytic effect.

Figure 4 and Figure 5 display the proposed reaction/stabilizing mechanisms of the PolyU against hydrolytic degradation. Due to the ring tension of the aziridine unit, as well as the partial charge caused by the different electronegativities of oxygen, carbon and nitrogen, the functional group is prone to react with the partially negatively charged oxygen atom of the moisture. In this step, the aziridine ring opens, and a proton of water is transferred to the nitrogen atom of the aziridine ring, while the OH^−^ is added to the carbon atom of the opened ring. The resulting compound has further functional groups which can react with the moisture until reaction products remain which do not allow for any further reaction. Overall, an additive molecule can react with moisture in several steps, which explains the high stabilizing effect of the aziridine-based additive. The reaction with the carboxyl end group follows a similar scheme; here, too, the partially negatively charged oxygen atom attacks the ring, causing it to open, and attaches to the carboxyl group. Thus, the carboxyl group is inhibited and cannot catalyze hydrolytic degradation reactions.

However, since aziridine-based additives have not yet been used as hydrolysis stabilizers in PLA or other hydrolysis-sensitive polymers, the stabilization mechanism was investigated, as described below, using nuclear magnetic resonance spectroscopy. For this purpose, the additive was reacted with the moisture and carboxylic acid groups, and ^1^H-NMR spectroscopy was performed to study the resulting products.

**Figure 6:**
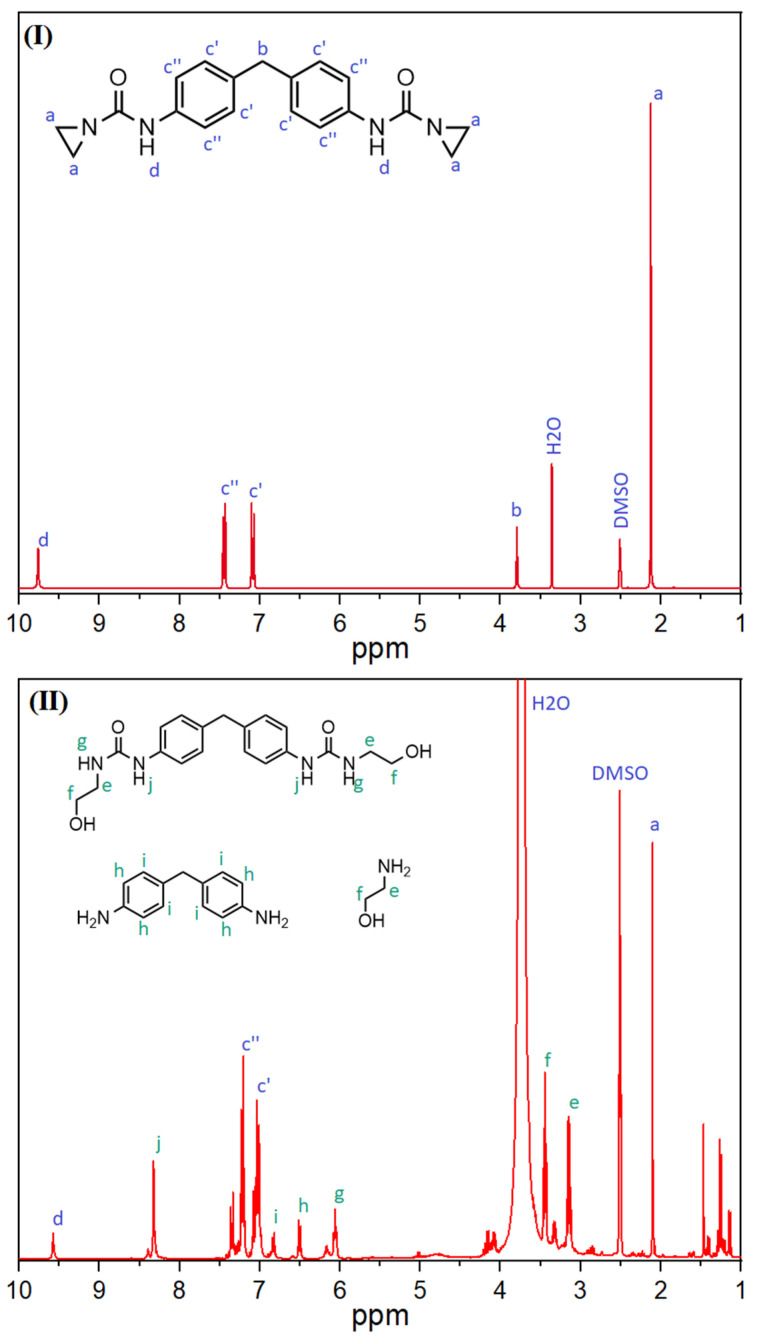
^1^H-NMR spectra of aziridine-based inhibitor before (**I**) and after the reaction with water (**II**). Blue letters indicate peaks of the initial substance, green letters indicate peaks generated by the reaction with moisture.

As can be seen in Figure 6, the proton peaks in ^1^H-NMR spectroscopy undergo a drastic change after the reaction of the aziridine-based inhibitor with water. In relation to the solvent peak, the signals of the protons from the aziridine ring (a; 2.1 ppm) are much smaller after the reaction, which can be attributed to the ring opening reactions resulting in new peaks (e and f) at different chemical shifts of 3.1 and 3.5 ppm. In addition, the proton on the nitrogen atom of the opened aziridine ring creates a new signal at around 6.0 ppm (g). The changed chemical environment also results in a shift of the proton of the amino group at 9.7 ppm (d) to 8.3 ppm (j). The consecutive reactions with moisture lead to a cleavage in the amino group, as described in Figure 4, forming 4,4’-diaminodiphenylmethane. Thus, there is also a shift in the peaks of the protons on the benzene ring from 7.1 and 7.3 ppm (c) to 6.5 (h) and 6.8 ppm (i), respectively. Therefore, it can be concluded that nuclear magnetic resonance spectroscopy proved the reaction of the aziridine-based hydrolysis inhibitor with moisture. Moreover, the newly formed peaks in the ^1^H-NMR spectrum could be assigned to the postulated degradation products.

**Figure 7:**
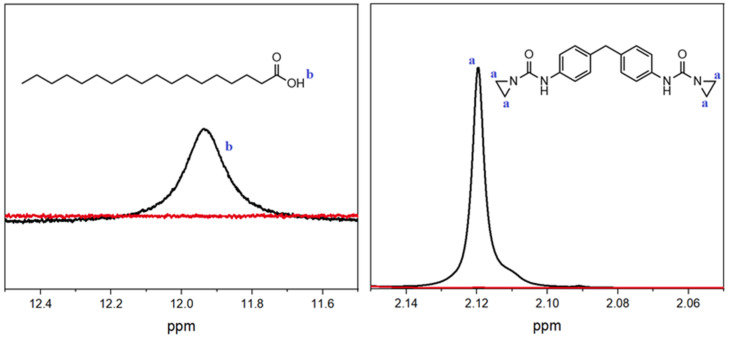
^1^H-NMR spectra of the stearic acid (l.) and PolyU (r.) mixture before (black line) and after (red line) reaction.

Furthermore, the reaction of the aziridine-based inhibitor with carboxylic acid groups was also demonstrated. For this purpose, the inhibitor and stearic acid were mixed and reacted in a solvent (DMSO-d6) at 150 °C. The peaks of the aziridine (2.1 ppm—a) and carboxyl groups (12.0 ppm—b) were analyzed (see Figure 7). The ^1^H-NMR spectra of the mixture revealed that the peaks that were clearly visible before the reaction (black line) disappeared afterwards (red line). In order to check whether the elevated temperature was not the only factor causing the groups to react, stearic acid alone was subjected to the same procedure. In this case, there was no change in the peak.

Additionaly FTIR spectra of the granules were recorded before and after hydrolysis to examine structural changes. However, only changes in the surface are observed through FTIR, but not in the bulk where the main degradation takes place. Therefore, the changes between the individual materials were only marginal (illustrated in Figures S1–S4).

**Figure 8:**
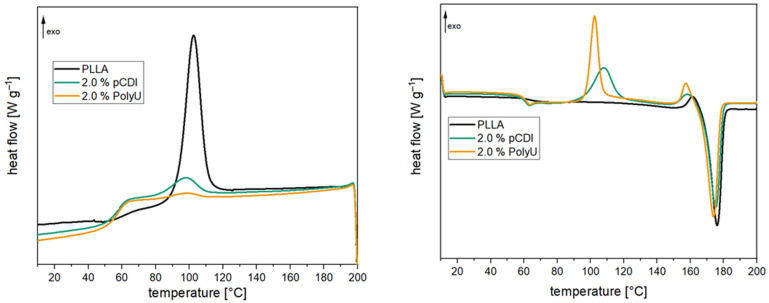
The 1st cooling (l.) and 2nd heating (r.) curves of PLA compounds with and without hydrolysis inhibitor (after compounding).

For the use of PLA in durable applications, a high heat deflection temperature is required in addition to good hydrolytic stability. A high degree of crystallization is required for this since amorphous PLA already softens at 60 °C [24]. In Table 1, the thermal properties of the compounds before and after aging are presented. The 1st heating cycle is influenced by the processing conditions or aging and drying after aging; it is excluded from the following discussion. Cooling (see Figure 8) shows that a significant crystallization peak develops due to the high l-content of the PLA used. The degree of crystallization is slightly above 30%. When the inhibitors are added, there is a significant change in the crystallization behavior. Both the polymeric carbodiimide and the aziridine-based inhibitor almost completely prevent the crystallization of the polymer chains, and the degree of crystallization is below 5%. Crystallization temperature is also slightly reduced by the inhibitors. During heating, on the other hand, there are only minor differences. The glass transition temperature is marginally lower, and the melt temperature is also somewhat reduced. However, these differences are not to be regarded as significant. As can be seen in Figure 8, the compounds with the inhibitors exhibit pronounced post-crystallization, which means that crystallization is not completely prevented but severely slowed down. The reason for this behavior is the high additive content. Since the additives are structurally incompatible with the PLA, they hinder the formation of crystallites [28]. The degree of crystallization decreases significantly, especially with higher addition quantities [32]. After two weeks of aging, a significant change is noticeable for the unstabilized PLA. On the one hand, the crystallization temperature is 7 °C higher than before, and on the other hand, the degree of crystallization is also significantly increased (+20%). This is due to the pronounced molecular weight reduction, which leads to significantly shorter chains. These are much more mobile and can, therefore, form crystallites earlier and more easily [56]. This is also noticeable for a significantly reduced melting temperature during heating, which is about 10 °C lower. In addition, no glass transition can be determined due to the high degree of crystallization. In the samples with hydrolysis inhibitor, however, no changes occur. The degree of crystallization remains unchanged at a low level, and the crystallization and melting temperatures do not change either. This, again, confirms the minimal changes in the polymer chain length and the good stabilizing effect of the inhibitors. The change in crystallinity is also visually noticeable. The PLA granules change from transparent to opaque after ageing. For the stabilized samples the changes are less pronounced (illustrated in Figure S5).

It can be concluded that a very good hydrolysis stabilizing effect can be achieved through the direct reaction of the inhibitors with the moisture and the acid groups. In addition, with the aziridine derivatives, a second inhibitor class was identified, which exhibits a stabilizing effect comparable to the carbodiimides. However, the negative effect of the inhibitors on the crystallization behavior was problematic. As described in the following subsection, an attempt was made to reduce the inhibitor content by adding a co-stabilizer.

### 3.2. Enhancement of the Hydrolytic Stability by Adding an Acid Regulator

**Figure 9:**
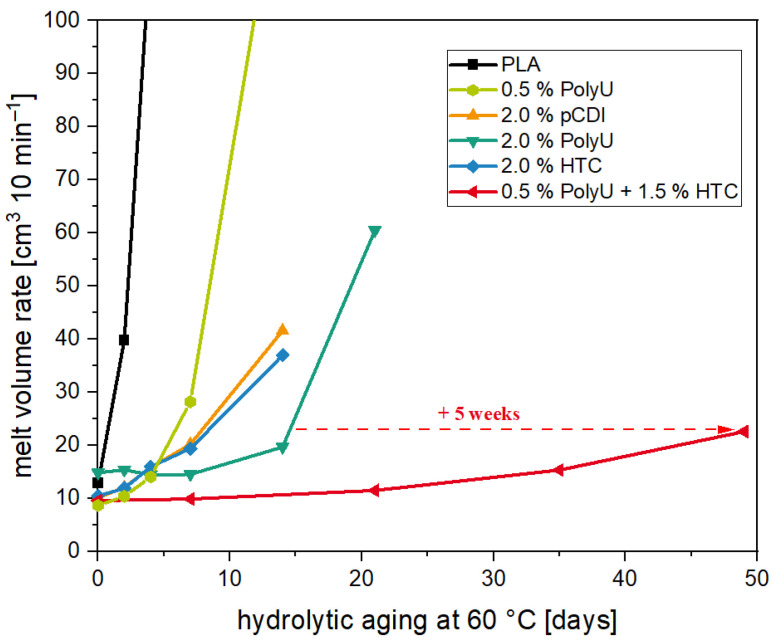
MVR values of aged PLA samples unstabilized (black) and stabilized with a novel stabilizer composition over the course of 49 days at a temperature of 60 °C.

The co-stabilizer used is an acid scavenger, specifically a hydrotalcite. The stabilizing effect of acid scavengers has been described in the literature before [48,49] but has not yet been investigated together with hydrolysis inhibitors. It was investigated whether a synergistic effect could be achieved through the different stabilization mechanisms of the two additive types. For this purpose, the aziridine derivative and the hydrotalcite were tested alone at 2.0% and at a 1:3 ratio at the same loading of 2.0%. Based on previous publications, it can be assumed that the inhibitor has the bigger effect. The aziridine derivative was also tested at 0.5% to have the same inhibitor content as in the 1:3 combination. The results of the accelerated hydrolysis aging are shown in Figure 9.

**Figure 10:**
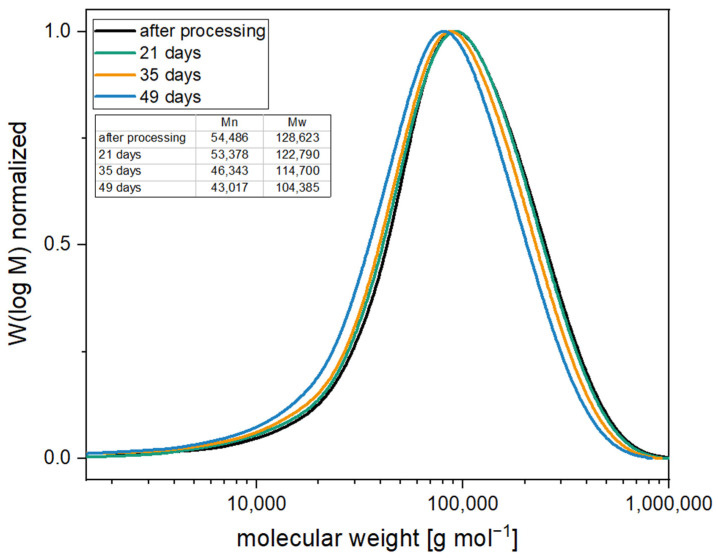
Molecular weight distribution of samples stabilized with 0.5% PolyU and 1.5% HTC under accelerated aging conditions (60 °C in water).

Accelerated aging revealed that the compounds with 0.5% PolyU exhibited only a short stabilization period. After only one week, the MVR increase was more than 300% (8.7 cm^3^ 10 min^−1^ → 28.2 cm^3^ 10 min^−1^), and after two weeks, the material had degraded to such an extent that no value could be measured. This can be attributed to the rapid additive consumption due to the low stabilizer content. As soon as the additive was used up, the degradation took place unhindered, since the migrating moisture was no longer reacted off. This behavior is also known for carbodiimides [29]. Even with a PolyU content of 2.0%, it was seen that beyond the two weeks, only a slight stabilizing effect occurred. Thus, after three weeks, the MVR was already approx. 500% (14.9 cm^3^ 10 min^−1^ → 60.6 cm^3^ 10 min^−1^) above the initial value. The addition of the hydrotalcite also contributed to a significant hydrolysis stabilization, although somewhat less than with the PolyU. The MVR almost doubled after one week (10.5 cm^3^ 10 min^−1^ → 19.4 cm^3^ 10 min^−1^), with further doubling after a total of two weeks (→ 37.0 cm^3^ 10 min^−1^). The change in MVR over the two weeks was comparable to the pCDI. The molecular weight loss of 20% (based on the number-average molecular weight) was also in a similar range. This correlates with studies on other acid-regulating additives, which also showed stabilization against hydrolytic chain degradation [45,46,47]. Poly(lactic acid) containing 2.0% hydrotalcite showed comparable hydrolytic stabilities over 14 days [49]. The stabilizing effect of the HTC is due to the acid-regulating effect. Thus, the basicity of the additive alters the pH of the moisture penetrating the polymer, reducing the rate of degradation reactions between the polymer chains and the moisture [48]. As postulated before, the most extended stabilizing effect resulted from the combination of the two additive types. Compared to the single additives, a clear synergistic effect was observed. While with 2.0% PolyU, the increase after three weeks was 500%, the MVR with the synergistic combination was only 15% (9.9 cm^3^ 10 min^−1^ → 11.5 cm^3^ 10 min^−1^). Even after five weeks of accelerated aging, the MVR increase was only 50% (→ 15.4 cm^3^ 10 min^−1^) and slightly more than doubled after seven weeks (→ 22.6 cm^3^ 10 min^−1^), while the unstabilized PLA showed a higher MVR and, thus, a stronger molecular weight degradation after two days in accelerated aging. Compared to the samples with an inhibitor only, the time until the start of degradation was prolonged by a factor of 3.5 (+ 5 weeks). Molecular weight studies (see Figure 10) confirm the excellent stabilizing effect of the synergistic combination of an inhibitor and acid scavenger. After seven weeks of accelerated aging, the molecular weight was reduced by just 20%.

**Figure 11:**
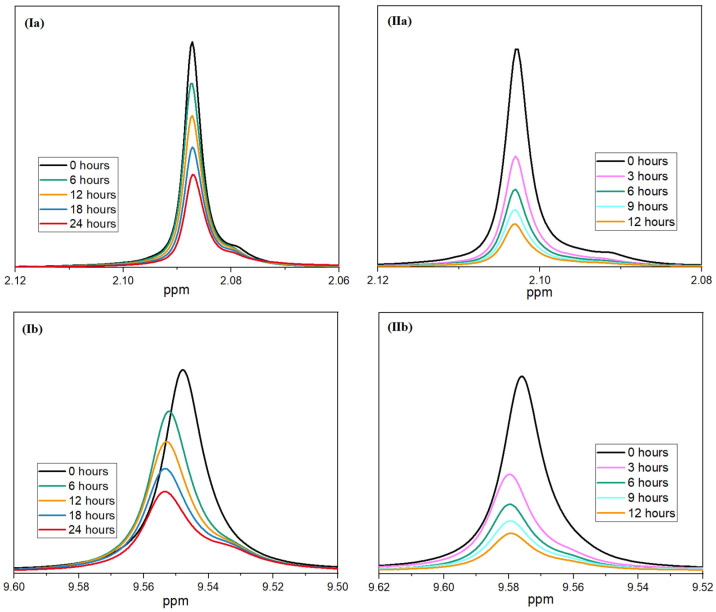
Rate of reaction of PolyU with moisture at different pH values (**I**—7.0; **II**—2.1); the signal of aziridine ring (**a**) and amino group (**b**) are displayed.

As described in the following, nuclear magnetic resonance spectroscopy was used to verify the synergistic effect of the aziridine derivative and the acidity regulator. It was assumed that the acid-regulating effect of the hydrotalcite not only slowed down the reaction of the moisture with the polymer chains, but also slowed down the reaction between the moisture and the inhibitor. This resulted in slower consumption, and the inhibitor could have a stabilizing effect during aging over a longer period of time. To investigate this, the PolyU was again reacted with moisture, but this time, at two different pH values: at pH 7 representing the state with an acidity regulator, and at pH 2.1, simulating the state without an acidity regulator. For this purpose, a 10% lactic acid solution was added to the water/NMR-solvent mixture. The samples were aged at 60 °C over a period of 24 and 12 h, respectively; ^1^H-NMR spectra were recorded at regular intervals. Figure 11 shows the signals for the aziridine ring (2.1 ppm) and for the amino group (9.6 ppm) as the reaction progressed. The slight shift in the peaks was likely caused by the decrease in pH. A clear dependence of the speed of the reaction of the two groups on the pH value can be seen in the figure.

**Figure 12:**
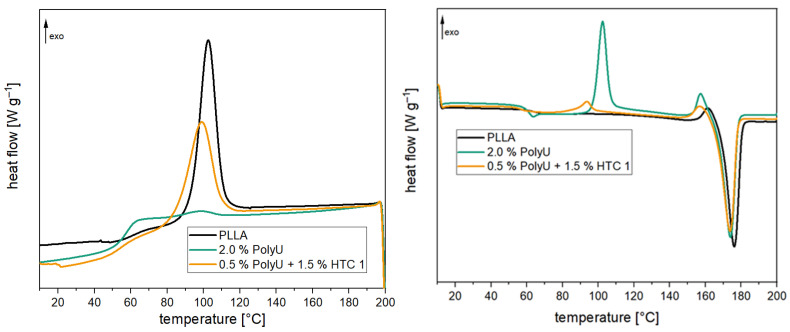
The 1st cooling (l.) and 2nd heating (r.) curves of PLA compounds with and without the stabilizer combination after compounding.

At a pH of 2.1, a much faster reduction of the two peaks can be seen. In particular, at the beginning, there is a rapid reduction. The peaks are reduced by half after only three hours. After 12 h, the two signals almost completely disappear. In comparison, the reaction of the two groups at a pH of 7 is much more uniform. The halving of the signal intensity can only be observed after approx. 18 h of reaction time. Even after 24 h of reaction, a distinct signal can still be observed for the aziridine ring and the amino group. This proves that the addition of the co-stabilizer can significantly reduce the rate of inhibitor consumption. Therefore, not only a significantly longer stabilizing effect is achieved, but also the loading of the inhibitor can be reduced. Furthermore, the negative effects on the crystallization behavior of PLA (see Figure 12 or Table 2) are minimized. The degree of crystallization increases from 0.9% (2.0% PolyU) to 25.6% (0.5% PolyU + 1.5% HTC) and is almost at the level of the unadditivated PLA (33.5%). The post-crystallization peak is also reduced accordingly during the subsequent heating cycle. Crystallization, glass transition and melting temperature remain unchanged.

## 4. Conclusions

A novel hydrolysis inhibitor class was identified based on an aziridine derivative. Compared to carbodiimides, which have already been widely investigated, they exhibit less degradation of the polymer chains during accelerated aging over 14 days at 60 °C in water. With the aid of nuclear magnetic resonance spectroscopy, it was possible to demonstrate that the aziridines react both directly with the moisture migrating into the polymer and with the carboxyl end groups of the poly(lactic acid). However, the negative effect on the crystallization behavior of the polymer chains can be problematic when using hydrolysis inhibitors (also in the case of carbodiimides). As a result, the polymer chains stay in an amorphous state, which results in a lower heat distortion temperature and, thus, may make PLA unsuitable for technical applications. In addition, the hydrolysis inhibitors are expensive compared to the polymer itself, which cause economical challenges. However, it was demonstrated that the combination of the inhibitor with an acid scavenger based on hydrotalcite resulted in a synergistic effect with respect to hydrolysis stabilization. It is concluded that due to the acid scavenger, the pH of the moisture migrating into the polymer can be altered. Therefore, not only the reaction between the moisture and the polymer chain is slowed down, but also the reaction between the hydrolysis inhibitor and moisture is slowed down, resulting in a delayed additive consumption and in an extension of the stabilizing effect. In an accelerated aging at 60 °C in water, it was proven that the duration until significant hydrolytic chain degradation occurs can be extended from two days (unstabilized PLA) to 14 days (2.0% inhibitor) and up to 49 days through the synergistic combination at a constant total additive content (0.5% inhibitor + 1.5% acid scavenger). By reducing the inhibitor content from 2.0% to 0.5%, the negative effect of the inhibitor on the crystallization behavior can be prevented, moreover, making the compound economically more attractive.

## 5. Patents

Hallstein, J.; Metzsch-Zilligen, E.; Pfaendner, R. Stabilizer composition, use of stabilizer composition, method for stabilizing condensation polymers against hydrolytic degradation, and hydrolysis-stabilized composition and molding or molded part consisting thereof. WO 2021/214207 A1, 22 September 2022.

## Figures and Tables

**Table 1:** Thermal properties of PLA compounds stabilized with hydrolysis inhibitors before (top) and after (below) hydrolytic aging at 60 °C for 14 days.

	1st Cooling		2nd Heating	
	T_cp_	X_cc_	T_g_	T_m_
PLLA	103 °C	33.5%	64 °C	175 °C
2.0% pCDI	98 °C	5.0%	61 °C	174 °C
2.0% PolyU	100 °C	0.9%	61 °C	173 °C
PLLA	110 °C	54.9%	--	165 °C
2.0% pCDI	99 °C	4.6%	61 °C	174 °C
2.0% PolyU	99 °C	5.6%	61 °C	173 °C

**Table 2:** Thermal properties of PLA compounds with different hydrolysis stabilizers.

	1st Cooling		2nd Heating	
	T_cp_	X_cc_	T_g_	T_m_
PLLA	103 °C	33.5%	64 °C	175 °C
2.0% pCDI	98 °C	5.0%	61 °C	174 °C
2.0% PolyU	100 °C	0.9%	61 °C	173 °C
2.0% HTC	99 °C	15.4%	61 °C	175 °C
0.5% PolyU + 1.5% HTC	99 °C	25.6%	58 °C	174 °C

## Data Availability

Dataset available on request from the authors.

## Supplementary Materials

The following supporting information can be downloaded at: https://www.mdpi.com/article/10.3390/polym16040506/s1, Figure S1: FTIR spectra of PLLA before and after hydrolysis. Figure S2: FTIR spectra of PLLA + 2.0% pCDI before and after hydrolysis. Figure S3: FTIR spectra of PLLA + 2.0% PolyU before and after hydrolysis. Figure S4: FTIR spectra of PLLA + 0.5% PolyU + 1.5% HTC before and after hydrolysis. Figure S5. Granules before (top) and after (bottom) hydrolysis.
