# Supplementary material for: Enhancing the Hydrolytic Stability of Poly(lactic acid) Using Novel Stabilizer Combinations
Source: Polymers (Basel). 2024 Feb 13;16(4):506. doi: 10.3390/polym16040506 (PMC10892727; doi:10.3390/polym16040506)
Supplement: Supplementary file 1 [file polymers-16-00506-s001.zip › polymers-2853631-supplementary.pdf]

polymers

MDPI

Supplementary information

Enhancing the Hydrolytic Stability of Poly(lactic acid) by Novel Stabilizer Combinations

Jannik Hallstein, Elke Metzsch-Zilligen and Rudolf Pfaendner *

Fraunhofer Institute for Structural Durability and System Reliability LBF, Division Plastics, 64289 Darmstadt, Germany.
jannik.hallstein@lbf.fraunhofer.de (J.H.);
elke.metzsch-zilligen@lbf.fraunhofer.de (E. M.-Z.)
* Correspondence: rudolf.pfaendner@lbf.fraunhofer.de

1. FTIR spectra of samples before and after hydrolysis

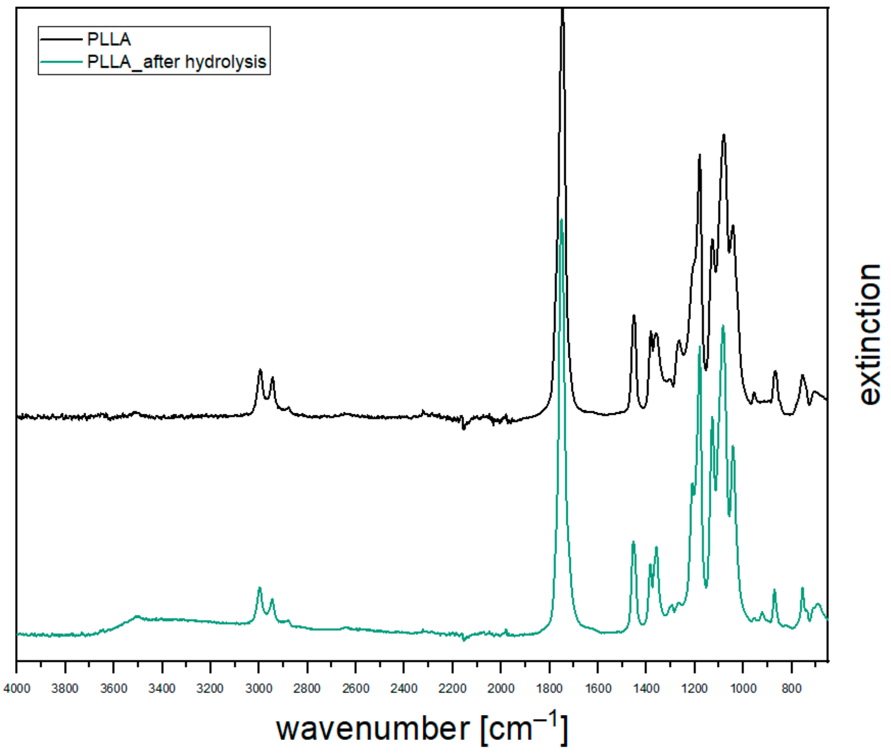

Figure S1. FTIR spectra of PLLA before and after hydrolysis

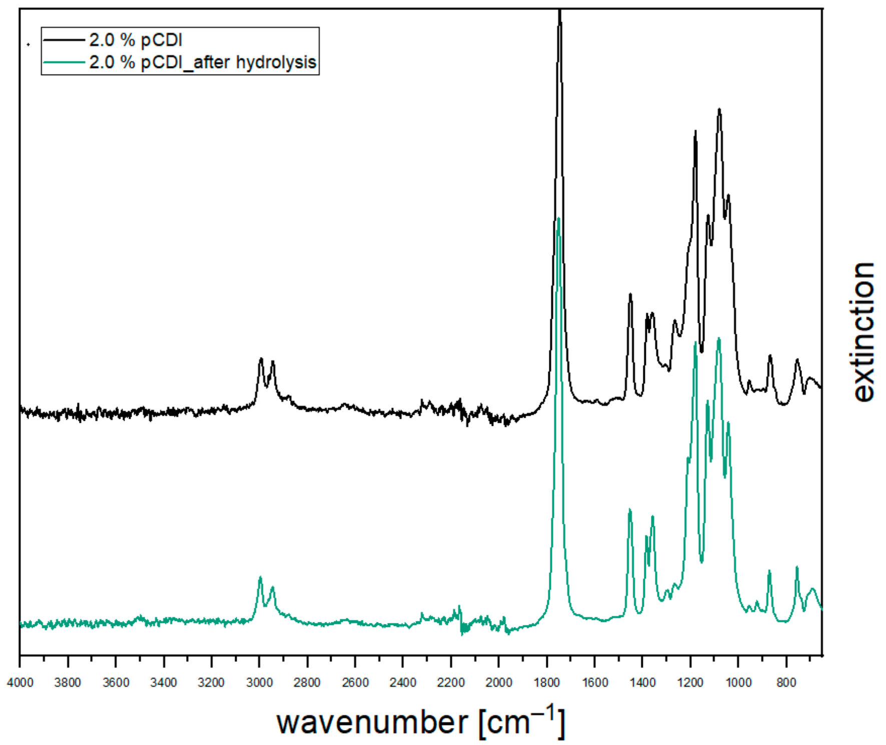

Figure S2. FTIR spectra of PLLA + 2.0 % pCDI before and after hydrolysis

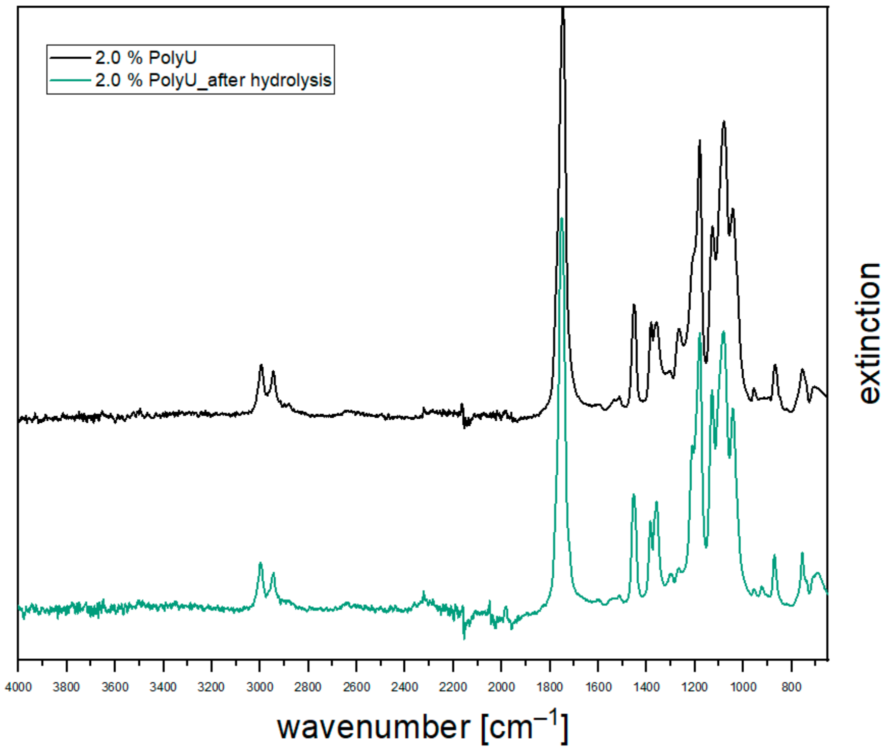

Figure S3. FTIR spectra of PLLA + 2.0 % PolyU before and after hydrolysis

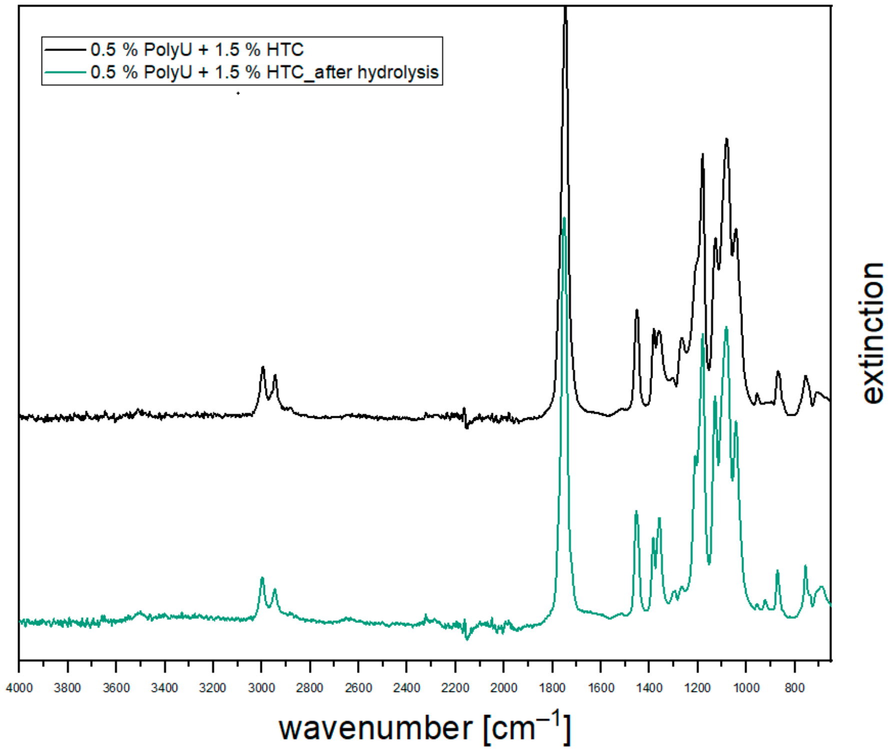

Figure S4. FTIR spectra of PLLA + 0.5 % PolyU + 1.5 % HTC before and after hydrolysis

2. Pictures of samples before and after hydrolysis

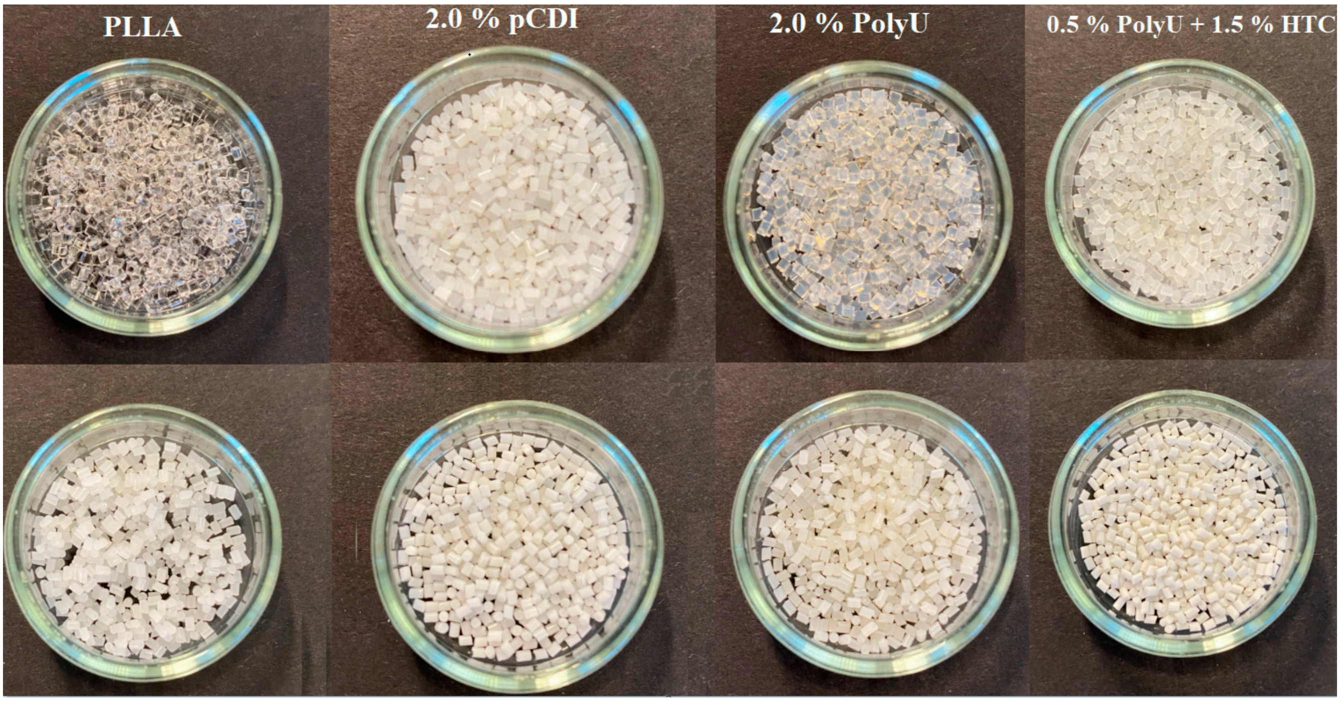

Figure S5. Granules before (top) and after (bottom) hydrolysis
